# Mitophagy in Cerebral Ischemia and Ischemia/Reperfusion Injury

**DOI:** 10.3389/fnagi.2021.687246

**Published:** 2021-06-08

**Authors:** Luoan Shen, Qinyi Gan, Youcheng Yang, Cesar Reis, Zheng Zhang, Shanshan Xu, Tongyu Zhang, Chengmei Sun

**Affiliations:** ^1^Zhejiang University-University of Edinburgh Institute, School of Medicine, Zhejiang University, Haining, China; ^2^VA Loma Linda Healthcare System, Loma Linda University, Loma Linda, CA, United States; ^3^Institute for Advanced Study, Shenzhen University, Shenzhen, China; ^4^Department of Neurosurgery, Xuanwu Hospital, Capital Medical University, Beijing, China

**Keywords:** mitophagy, mitochondrial dysfunction, ischemic stroke, ischemia/reperfusion injury (I/R injury), recanalization therapy, therapeutic window

## Abstract

Ischemic stroke is a severe cerebrovascular disease with high mortality and morbidity. In recent years, reperfusion treatments based on thrombolytic and thrombectomy are major managements for ischemic stroke patients, and the recanalization time window has been extended to over 24 h. However, with the extension of the time window, the risk of ischemia/reperfusion (I/R) injury following reperfusion therapy becomes a big challenge for patient outcomes. I/R injury leads to neuronal death due to the imbalance in metabolic supply and demand, which is usually related to mitochondrial dysfunction. Mitophagy is a type of selective autophagy referring to the process of specific autophagic elimination of damaged or dysfunctional mitochondria to prevent the generation of excessive reactive oxygen species (ROS) and the subsequent cell death. Recent advances have implicated the protective role of mitophagy in cerebral ischemia is mainly associated with its neuroprotective effects in I/R injury. This review discusses the involvement of mitochondria dynamics and mitophagy in the pathophysiology of ischemic stroke and I/R injury in particular, focusing on the therapeutic potential of mitophagy regulation and the possibility of using mitophagy-related interventions as an adjunctive approach for neuroprotective time window extension after ischemic stroke.

## Introduction

Stroke is a sudden onset of cerebral blood circulation disorders caused by cerebral infarction or hemorrhage (Shi et al., 2021). Depending on the area of the brain affected, patients may present with different symptoms, among which the most common ones are the acute onset of weakness in one-side of the body and a reduced speaking ability (Nor et al., 2005). Stroke is the fifth leading cause of death according to American Heart Association (AHA). Around 795,000 people suffer from either new or recurrent stroke each year (Virani Salim et al., 2020). There are two major types of stroke: ischemic stroke and hemorrhagic stroke. In ischemic stroke, blood flow is blocked by thrombosis formed around the ruptured atherosclerotic plaques in the artery, while hemorrhagic stroke usually results from bleeding induced by blood vessel rupture.

Ischemic stroke accounts for about 87% of all stroke cases. Thus it is under great attention in research and clinical practice. Blocked blood flow leads to the lack of oxygen and nutrients, triggering an ischemic cascade in the brain. The production of adenosine triphosphate (ATP) would be disrupted, which is often a lethal situation to vulnerable brain cells that are highly energy-dependent. In detail, failure in ATP generation can result in the weakened activity of ATP-dependent ion channels, including sodium channels, thus causing intracellular hyperosmolarity (Deb et al., 2010). Also, increased anaerobic respiration during ischemia produces byproduct lactic acid, leading to metabolic acidosis. Severe alternations in the ion balance can cause cytotoxic edema, disrupt the glutamate receptor activity, which eventually damages DNA and structural proteins or even leads to cell deaths (Nishizawa, 2001; Deb et al., 2010). Irreversible neuropathological changes in neurons usually occur within 20–30 min after ischemia (Ordy et al., 1993).

Reperfusion treatments which aim at restoring blood flow and oxygen to the ischemic area before neuronal damage, are the primary management for ischemic stroke patients in the clinic. Intravenous tissue plasminogen activator (IV tPA) is the only FDA-approved thrombolytic agent for treating acute stroke. Previous evidence suggests it only shows significant clinical improvement when given within 3 h after ischemia (Kwiatkowski et al., 1999). Another randomized trial study performed in 2017 indicated that intravenous therapies within 6 h still benefit over safety concerns (Berkhemer et al., 2014). Mechanical thrombectomy, a surgical procedure to remove thrombosis from arteries, is another commonly used reperfusion therapy. Thrombectomy is efficient in reducing post-stroke disability, though its efficacy and safety can only be ensured within 8 h after the stroke onset (Jovin et al., 2015). In recent years, two high-quality clinical trials focusing on delayed recanalization indicate that reperfusion treatment given at 24 h or even later after the stroke onset still show some improved prognosis in selected patients, thus extending the therapeutic window to 24 h in specific patient populations (Ragoschke-Schumm and Walter, 2018). However, all current treatments have the major limitation of increasing the risk of intracranial hemorrhage (ICH) when given outside the therapeutic window, which can further damage the brain tissue. This subsequent injury following reperfusion therapy is termed as ischemia/reperfusion (I/R) injury, a process that involves reoxygenation-induced reactive oxygen species (ROS) production, calcium overload, and tissue damage. Therefore, extending the reperfusion time window after ischemic stroke while providing neuroprotection is extremely important for disease management.

Autophagy is a natural process that degrades unnecessary or damaged organelles and proteins to maintain cellular homeostasis. Autophagy can be activated after ischemic stroke when brain cells are exposed to the risk of oxygen and nutrients deficiency. To be more specific, oxygen-glucose deprivation stimulates the increase in AMP/ATP ratio, which is an activator for the AMPK pathway (Oakhill et al., 2011; Jiang et al., 2018). Upregulation of the AMPK pathway can thus initiate autophagy via direct activation of the ULK complex through phosphorylating of Ser 317 and Ser 777, or indirect activation of ULK through inhibiting the activity of mTOR, as mTOR suppresses Ulk1 activation by phosphorylating Ulk1 Ser 757 and disrupting the interaction between Ulk1 and AMPK (Egan et al., 2011; Kim J. et al., 2011). Previous researches shows controversial results regarding the role of autophagy after ischemic stroke. Some studies show that autophagy provides neuroprotection and improves clinical outcomes by significantly reducing ischemic damage of neurons, glia, and endothelial cells (Papadakis et al., 2013; Jiang et al., 2015; Dai et al., 2017). Meanwhile, other findings suggest that excess autophagy might be harmful to brain cells (Li et al., 2017; Mo et al., 2020). To sum up, despite the controversial evidence, it is generally agreed that moderate autophagy is protective, while excessive autophagy may contribute to cell deaths during ischemia (Mo et al., 2020). Ischemic preconditioning (IPC), a strategy that uses short periods of vascular occlusion and reperfusion to prevent fetal ischemic events and recanalization, can activate the neuroprotective program in the brain via triggering adaptive autophagy targeting damaged organelles and alleviating oxidative stress in the acute ischemic stroke (Yang et al., 2020; Ajoolabady et al., 2021). In addition, cerebral ischemia postconditioning, which reduces maladaptive autophagy by applying short periods of reperfusion interrupted by ischemia at the beginning of recanalization, has been induced to suppress reperfusion injury, indicating its protective role in treating ischemic stroke (Vinten-Johansen, 2017; Ajoolabady et al., 2021).

Mitophagy, a type of selective autophagy, can remove dysfunctional mitochondria. Mitochondria play a central role in cellular energy production, calcium homeostasis maintenance and ROS regulation. Mitochondrial dysfunction can increase oxidative stress and cellular damage (Liu, 1999; Indo et al., 2007). Mitophagy mainly work as a mitochondrial quality control through the clearance of damaged mitochondria. In mammals, dysfunctional mitochondria can be cleared either via PINK1-Parkin dependent ubiquitination pathway or via the activation of mitophagy receptors, thus reducing ROS generation from mitochondria (Lemasters, 2005) and protecting cells against unfavorable niche (Huang et al., 2011). Under the condition of ischemic stroke, malfunctioned mitochondria increase the release of pro-apoptotic factors including cytochrome c, to induce cell deaths in the affected area (Jürgensmeier et al., 1998; Lemasters, 2005). It is worth note that mitophagy may have different effects during the first ischemic phase and later the reperfusion phase. Studies have indicated that mitophagy exerts its protective role mainly during the reperfusion phase (Kumar et al., 2016).

In recent years, the role of mitophagy in acute stroke has been extensively studied. Most studies indicate a neuroprotection role of mitophagy in alleviating reperfusion injury through multiple mechanisms. This review summarizes the role of mitophagy in ischemic stroke and I/R injury, proposing mitophagy-related interventions as an adjunctive approach for ischemic stroke management. Updates regarding delayed recanalization and the potential involvement of mitophagy in it were also discussed.

## Mitochondria and Mitophagy

**FIGURE 1:**
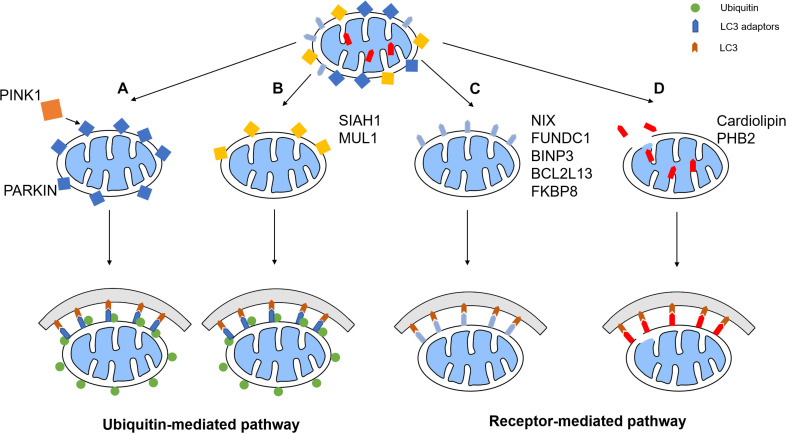
Mitophagy pathways. The mitophagy pathways can be generally divided into two types: ubiquitin-mediated pathway and receptor-mediated pathway. **(A)** PINK1 phosphorylates the E3 ubiquitin ligase Parkin, which promotes the ubiquitination of OMM proteins. Some of these proteins recruit LC3 adaptors, which induces mitophagy. **(B)** Other E3 ligases such as SIAH1 and MUL1 also has similar pathways. **(C)** Some mitophagy receptors such as NIX, FUNDC1, BNIP3, BCL2L13, and FKBP8, are upregulated or activated under certain conditions, therefore recruiting phagophore to initiate mitophagy. **(D)** IMM receptors including cardiolipin and PHB2 recruits phagophore by interacting with LC3 to promote mitophagy.

Mitochondria are an important organelles that is mainly responsible for energy production. However, damaged mitochondria release harmful ROS and other oxidants, such as H_2_O_2_ and peroxynitrite, into the cytoplasm and cause damage to the proteins, nuclear acid, and membranes (Zhou et al., 2011). Worse over, cytochrome c, a mitochondrial intermembrane space protein, will be released under severe mitochondrial damage, which triggers caspase cascade and finally apoptosis (Ott et al., 2002). Therefore, rapid degradation of damaged mitochondria is necessary for cell survival. Mitophagy is a process during which damaged or aging mitochondria are selectively wrapped by phagophores and undergo lysosomal degradation to maintain cell homeostasis and prevent cell apoptosis. Mitophagy starts with the formation of the phagophore, a membrane structure isolated from the endoplasmic reticulum. Phagophore then recognizes damaged mitochondria through LC3 adaptors or LC3 receptors and engulfs damaged mitochondria for autosomal degradation. Currently, the mitophagy pathways consist of two major types: ubiquitin-mediated pathway and receptor-mediated pathway (Figure 1).

### Ubiquitin-Mediated Pathway

The PTEN-induced putative kinase protein 1 (PINK1) and Parkin-mediated ubiquitination pathway are some of the most well-characterized mitophagy mechanisms. In healthy mitochondria, PINK1, a serine/threonine kinase, is imported from the cytoplasm continuously, and undergoes cleavage by the mitochondrial proteases mitochondrial-processing peptidase (MPP) and presenilin associated rhomboid like (PARL) (Greene et al., 2012). Upon ischemic stroke, the mitochondria membrane is depolarized, which prevents the import of PINK1 and results in the accumulation of PINK1 on the mitochondrial membrane. As a result, the kinase activity of full-length PINK1 induces phosphorylation of E3 ligase Parkin, which activates the enzymatic function of Parkin and leads to ubiquitination of several mitochondrial proteins (Kazlauskaite et al., 2015). Meanwhile, PINK1 phosphorylates ubiquitin and results in binding of phosphorylated ubiquitin and Parkin, where the ligase activity is further improved (Koyano et al., 2014; Kazlauskaite et al., 2015).

Once Parkin is activated, it conjugates ubiquitin moieties onto the OMM proteins and thereby mitophagy is induced. Some ubiquitinated mitochondrial proteins, such as MFN1, are degraded, which is essential for the mitochondrial fission and mitophagy (Tanaka et al., 2010). Other ubiquitinated proteins recruit autophagy adaptor proteins such as optineurin (OPTN) and nuclear dot protein 52 (NDP52), which anchors the marked mitochondria to autophagosome by its LC3-interacting region (LIR) motifs, thereby mitophagy is initiated (Lazarou et al., 2015). Moreover, PINK1 can recruit OPTN and NDP52 independently of parkin, which subsequently recruits several autophagy initiation factors, such as unc-51-like autophagy activating kinase 1 (ULK1), for mediating phagophore synthesis and elongation (Wong and Holzbaur, 2014; Lazarou et al., 2015).

Several other E3 ligases have also been discovered to initiate mitophagy. Some mechanisms are related to PINK1/Parkin mediated mitophagy and some are independent of Parkin. For example, overexpression of mitochondrial ubiquitin ligase 1 (MUL1) mediates the degradation of mitofusin by ubiquitination, which rescues the PINK1/parkin mutant phenotype (Yun et al., 2014). In mature neurons, MUL1 is also important for the contact of ER and mitochondria and the absence of it impairs the Ca^2+^ homeostasis in mitochondria and reduces the intake of Ca^2+^ from ER. This leads to activation of calcineurin, which activate Drp1 and therefore induce mitochondria fission. Fragmented mitochondria lose their membrane potential, and PINK1/Parkin mediated mitophagy is induced (Puri et al., 2019). Another E3 ligase, seven *in absentia* homolog (SIAH)-1, is recruited by synphilin-1 when full-length PINK1 is present. SIAH-1 promotes mitophagy through ubiquitination of mitochondrial proteins independently of Parkin (Szargel et al., 2016).

In addition to the benefits of PINK1-Parkin mediated ubiquitination, deubiquitinases are essential for correct mitophagy. The deubiquitination of Parkin is carried out directly by ubiquitin-specific peptidase 8 (USP8) (Durcan et al., 2014), while USP15 deubiquitinates the substrates of Parkin to inhibit mitophagy (Cornelissen et al., 2014). Several other deubiquitinases, such as USP30, USP35, and USP33 (Bingol et al., 2014; Wang Y. et al., 2015; Niu et al., 2020), counteract ubiquitin-mediated mitophagy by removing ubiquitin chains from the mitochondrial membrane. Therefore, a fine-tuned balance between ubiquitination and deubiquitination is established for the regulation of mitophagy.

### Receptor-Mediated Pathway

An alternative pathway of mitophagy is through mitophagy receptor signaling. Multiple mitophagy receptors are currently identified in mammalian cells (Ren et al., 2018), which contains a least one LIR for the direct binding of autophagy mediator LC3 and the subsequent phagosome engulfment.

A critical receptor in the turnover of mitochondria in erythrocytes is an OMM protein named BCL2 interacting protein 3 like (BINP3-L, also known as NIX) (Sandoval et al., 2008). It is transcriptionally upregulated during erythrocytes maturation to clear mitochondria. In conditions of hypoxia, the BINP3-L is induced along with its homolog, BNIP3, promoting mitophagy through OPA1 disassembly and DRP1 recruitment, which is transcriptionally regulated by forkhead box O3 (FOXO3) and hypoxia-inducible factor (HIF), thereby inducing mitochondrial fission and inhibiting mitochondrial fusion (Sowter et al., 2001; Mammucari et al., 2007). Moreover, its binding affinity to LC3 is further improved by the phosphorylation of the LIR under stress conditions (Rogov et al., 2017).

Another essential mitophagy receptor is the FUN14 domain containing 1 (FUNDC1), which mediates mitophagy under hypoxic conditions (Liu et al., 2012). FUNDC1 activity is regulated by its phosphorylation state. In non-stress conditions, it is suppressed by phosphorylation of Src at Tyr18 and casein kinase II (CK2) at Ser13 (Chen et al., 2014). Under hypoxia, PGAM5 phosphatase dephosphorylate FUNDC1, which activates the LIR motif on FUNDC1 and induce mitophagy. Moreover, FUNDC1 recruits Drp1 and disrupts its physical association with OPA1 under stress (important for mitochondria dynamic), thereby inducing mitochondrial fission and inhibiting mitochondrial fusion (Chen et al., 2016).

Additionally, there are multiple other mitophagy receptors. On the outer mitochondrial membrane, BCL 2 Like 13 (BCL2L13) and FKBP prolyl isomerase 8 (FKBP8) have been shown to mediate mitophagy by binding LC3 via the LIR motif independently of Parkin (Murakawa et al., 2015; Bhujabal et al., 2017). Some receptors also locate in the IMM, such as prohibitin 2 (PHB2) and cardiolipin. Once the OMM is depolarized or damaged, PHB2 will interact with LC3 to directly promote mitophagy (Wei et al., 2017). However, the depletion of PHB2 upon OMM rupture destabilizes PINK1 through the activation of PARL and therefore leads to cleavage of full-length PGAM5 (Yan et al., 2020). This abolishes PGAM5-involved PINK1 stabilization and thereby inhibits PINK1/Parkin-dependent mitophagy. Recently, cardiolipin, a phospholipid, has also been identified as a mitophagy receptor, whose primary synthesis is conducted in the IMM. When encountering OMM rupture, cardiolipin is released to the OMM and interacts with LC3, triggering a signaling cascade that results in engulfment of the mitochondria (Chu et al., 2013).

### Mitochondria Dynamics and Its Relationship With Mitophagy

To adapt to the external environment, mitochondria fuses both the inner and outer membranes or undergo fission and separate into several mitochondria. These two essential processes in mitochondria dynamics are termed fusion and fission. When confronting cellular stress, fusion is promoted to ensure energy production by repairing partially damaged mitochondria (Youle and van der Bliek, 2012). On the other hand, fission is necessary for mitophagy since it enables the separation of depolarized mitochondria, allowing the preservation of “the healthy part” in mitochondria and reduces unnecessary loss during mitophagy. Depending on the quality of mitochondria, either fusion or fission will be activated along with the inhibition of the other (Twig et al., 2008).

In mammalian cells, MFN1, MFN2, and OPA1, which are GTPases, mediate the fusion of the mitochondria (Wu et al., 2019). These proteins are often modified post-transcriptionally to control their potency. For MFN1, extracellular regulated kinase (ERK) can phosphorylate it at Thr562 to suppress fusion. MFN1 can also be ubiquitinated by MARCH5 for degradation (Park et al., 2014). Mitogen-activated protein kinase 8 (MAPK8, also known as JNK) phosphorylates MFN2 at Ser27 under stress for subsequent ubiquitination by E3 ligases Parkin (Gegg et al., 2010), HUWE1 (Leboucher et al., 2012), and mitochondrial ubiquitin ligase membrane-associated RING-CH (MARCH5) (Sugiura et al., 2013). MFN1 and MFN2 can be deubiquitinated by USP30, where inhibition of it will lead to non-degradative ubiquitination of MFN1/2 (Yue et al., 2014). For OPA1, it is regulated by changes in the protease activity of YME1L and OMA1, which is responsive to intramitochondrial signals (Griparic et al., 2007; Head et al., 2009).

The mitochondrial fission is regulated mainly by a cytosol protein, DRP1, whose recruitment is mediated by mitochondrial fission factors such as MFF. DRP1 can be phosphorylated by protein kinase A at Ser637 and Ser656, which inhibits its activity. Dephosphorylation of DRP1 is mediated by the calcium-dependent protein phosphatase calcineurin or by protein phosphatase 2A (PP2A) for enhanced fragmentation under stress (Chang and Blackstone, 2007; Cribbs and Strack, 2007). Moreover, energy-sensing adenosine monophosphate (AMP)-activated protein kinase (AMPK) phosphorylates MFF under energy stress, which recruits Drp1 and accelerate mitochondrial fission (Toyama et al., 2016).

Mitophagy is closely interrelated with mitochondrial dynamics as multiple mitophagy proteins are found to promote fission and facilitate mitophagy. For instance, phosphorylated Parkin can ubiquitinate MFN1 and MFN2 for degradation, which decreases mitochondrial fusion and enhance fragmentation, leading to the initiation of mitophagy (Tanaka et al., 2010). During mitophagy, MFN2 is also phosphorylated by PINK1 to recruit Parkin for further mitophagy (Chen and Dorn, 2013).

## Pathophysiology of Ischemic-Reperfusion Injury (Figure 2)

### Clinical Classification of Ischemic Stroke

Ischemic stroke, also known as cerebral ischemia, is the significant type of all stroke cases. This disease occurs when blood clots or plaques block or narrow the brain arteries. Depending on the pathological condition, ischemic stroke can be divided into several subtypes: Intracranial arterial stenosis, acute arterial occlusion, and chronic arterial occlusion. Intracranial arterial stenosis refers to the narrowing of arteries caused by the formation of fatty deposits called atherosclerotic plaques and the concurrent thickening of vessel walls. In Intracranial arteries, including middle cerebral arteries, basilar artery, carotid arteries, and intracranial vertebral arteries, narrowed blood vessels can significantly reduce blood flow, leading to an ischemic event (Chimowitz et al., 2005; Banerjee and Chimowitz, 2017). A systematic analysis focusing on the role of intracranial atherosclerosis in ischemic stroke indicates that atherosclerosis-inducing stenosis graded higher than 30% can be a cause of fatal brain infarction (Mazighi et al., 2008). The atherosclerotic plaque is thrombogenic. Once its cap is ruptured, an unstable clot can be formed to narrow or completely occlude the arteries. The blood clot that blocks the affected site can form locally or originate elsewhere, such as in the heart, and embolize through the circulatory system. Rupture of plaques and clot embolisms is usually linked with acute arterial occlusion, manifesting stroke symptoms within hours (Malhotra et al., 2017). The occlusion can also be chronic (lasting more than 4 weeks) if the brain alters the cerebral hemodynamics and compensates the blood flow by building a collateral circulation in response to the reduced arterial blood supply (Sundaram et al., 2017). In that case, with sufficient collateral compensation, the disease can be asymptomatic and benign (Powers et al., 2000); Chronic occlusion without enough compensation from collateral circulation might still result in chronic cerebral hypoperfusion, leading to ischemic infarction. In some cases, patients with chronic occlusion may spontaneously recanalize over a long time (more than 3 months) (Delgado et al., 2015).

**FIGURE 2:**
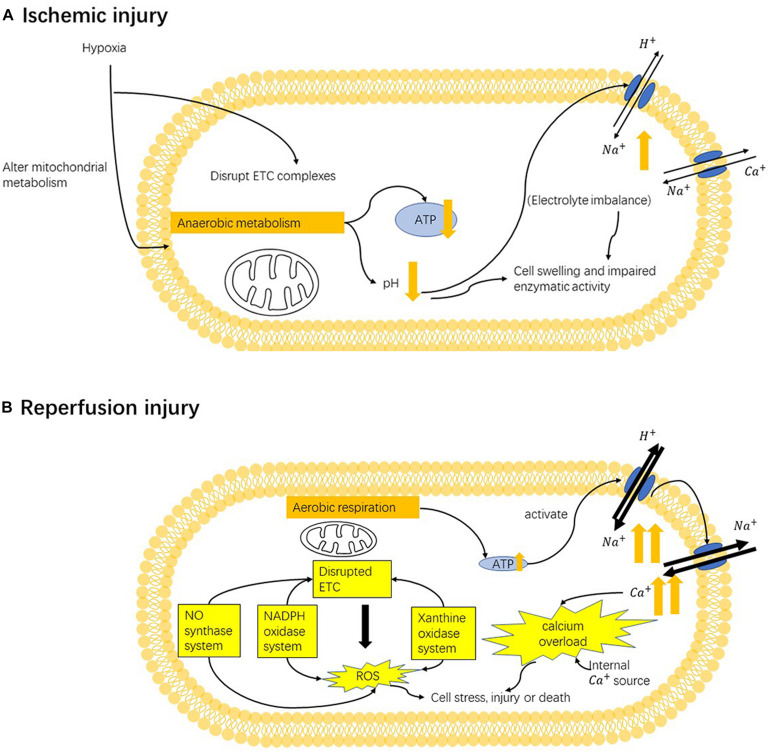
Mechanism of ischemia-reperfusion injury. **(A)** In the ischemic state, hypoxia (oxygen deficiency) converts mitochondrial metabolism from aerobic to anaerobic, resulting in lower ATP generation. Anaerobic respiration also induces metabolic acidosis and leads to the activation ion-exchange channels. Altered electrolyte imbalance can cause cell swelling and impair enzymatic activity. Moreover, under hypoxia, the mitochondrial electron-transport chains (ETCs) are also disrupted. **(B)** In the reperfusion state, mitochondrial damage and cellular alternations caused by previous hypoxia can stimulate the production of reactive oxygen species (ROS) from multiple sources: the NADPH oxidase system, nitric oxide synthase system, xanthine oxidase system and mainly mitochondrial electron-transport chains. Such enhanced oxidative stress can target mitochondria ETC and cause further damage ETC, causing more ROS production. Oxidative stress can cause secondary cellular damage. Restoration of oxygen can induce the overactivation of pumps that are inhibited by ischemia-induced ATP deficiency, which rapidly triggers the influx of sodium ions and later calcium ions. Altering calcium homeostasis causes cellular damage, stimulates ROS production and induces cell death by triggering the opening of mPTP and activating apoptotic pathway.

### Management of Ischemic Stroke

Thrombolytic agents and recanalization procedures are developed as reperfusion strategies to recover the blood flow in affected arteries. Usually, different therapeutic approaches are given to these three subtypes of stroke in the clinic setting. Due to technical limits, severe stenosis and acute occlusion of arteries are difficult to distinguish accurately (Clevert et al., 2006). Still, the correct diagnosis can be beneficial for optimal treatment and a better prognosis. Intravenous thrombolysis is the only approved therapy for AIS patients and can be given within 3 h of symptom onset. However, clinical outcomes of thrombolytic medical treatment alone for patients with severe stenosis and occlusion have shown worse than expected prognosis and less efficacy (Mokin et al., 2012). Clinical trials focusing on lysis of clots suggest that intravenous thrombolytic therapy alone has a low recanalization rate of only 30–40% among patients (Chen et al., 2012). Another analysis of clinical outcomes of intravenous thrombolysis for internal carotid artery occlusion suggests the rate of favorable outcomes be 25% (Mokin et al., 2012). Revascularization treatments, including stenting or endarterectomy, have thus been advised for patients with moderate or severe stenosis. Compared with intravenous thrombolysis, thrombectomy recipients have a significantly reduced incidence of ipsilateral stroke, meaning a better prognosis. Arterial therapies also achieve a better outcome in patients with acute occlusion (Mokin et al., 2012). However, in the clinic setting, endarterectomy is not considered as an option by many in treating complete ICA occlusion since this operation is still technically challenging to perform in preventing postoperative thrombus generation and maintaining a good prognosis (Kao et al., 2007; Chen et al., 2012; Faggioli et al., 2013). Until now, the search for effective treatments for chronic occlusion continues. Medical treatment like anti-platelet aggression drugs or intravenous tissue plasminogen activator can be given to patients to decrease the risk of stroke. Surgical approaches like endarterectomy and stenting can also be used in treating chronic occlusion, though they still show some seeming drawbacks. Like in acute occlusion, endarterectomy might fail in cases with complex clot organization, and the successful rate of recanalization only achieves 40% in patients with chronic occlusion (Thompson et al., 1986; Xu et al., 2018). Hypoperfusion still occurs in patients who have failed to restore blood flow in recanalization therapies, which is presumed to result in the recurrence of ischemic events (Grubb et al., 1998). Also, in the process of stenting, the clot may detach when the stent is released, blocking the intracranial artery and may therefore causing post-operative complications (Xu et al., 2018).

### Ischemia-Reperfusion Injury

In patients receiving recanalization therapies, sudden restoration of blood flow can sometimes be harmful, leading to the so-called ‘reperfusion injury.’ I/R injury refers to the tissue reoxygenation injury caused by the sudden return of blood supply to formerly ischemic or anoxic tissues. During the ischemia phase, the blood supply below standard functional requirements will cause deficiencies in oxygen and nutrients, leading to metabolic disturbances (Irie et al., 2014) and inflammatory response (Jin et al., 2013) in affected areas. Restoration of blood flow thus has been considered as a fundamental treatment to preserve tissue function. Loads of research and clinical trials on reperfusion treatments have shown that reperfusion therapies, including intravenous thrombolytic agents and endovascular interventions like mechanical thrombectomy, are relatively safe and can help with the recovery of acute ischemic stroke (AIS) patients when given inside a narrow time window (Kwiatkowski et al., 1999; Lees et al., 2010; Berkhemer et al., 2014; Jovin et al., 2015). However, reperfusion might also cause secondary injury in the previously ischemic tissues since the resupply of nutrients and oxygen can trigger considerable ROS production and accumulation and meanwhile alters calcium homeostasis, resulting in excessive oxidative stress and local inflammation. Such cellular changes cause cell damage and may activate the cell death pathway in the former ischemic tissues.

### Process and Mechanisms of I/R Injury (Figure 2)

#### Excessive Oxidative Stress Plays a Critical Part in I/R Injury

Oxidative stress is a disturbance in the balance between free radicals and antioxidant ability, and it often occurs when the production of ROS surpasses antioxidant defense. In the ischemic stage, obstructed blood flow with less oxygen and nutrient supply induces a shift in mitochondrial metabolism from aerobic to anaerobic, thus producing a lower concentration of ATP and antioxidative agents in cells. Later return of blood flow to the ischemic tissue can cause the reactivation of mitochondrial aerobic respiration and thus increase the production of ROS. Because of the decreased level of antioxidative agents, oxidation exceeds antioxidation during the reperfusion period, thus causing increased oxidative stress. Enzyme systems, including xanthine oxidase system, the NADPH oxidase system, nitric oxide (NO) synthase system, and mitochondria electron transport chain, are involved mainly in the occurrence of oxidative stress.

In normal cells, purine metabolism initiates from converting ATP to inosine with the participation of deaminases and nucleotidases, followed by its further transformation into hypoxanthine. Oxidation of hypoxanthine to xanthine and xanthine to uric acid later occurs, and xanthine dehydrogenase (XDH) and xanthine oxidase (XOD) separately function in these two oxidation processes. XDH utilizes NAD^+^ as an electron acceptor to produce NADH, and ischemia state can induce its shift to XOD that uses O_2_ as an acceptor (Kinuta et al., 1989). Restoration of blood flow and oxygen can stimulate the oxidation process in purine metabolism. Since the level of XOD is previously promoted, the formation of uric acid in the reperfusion phase is accompanied by the production of highly reactive superoxide anion (O2^–^). Superoxide can be later shifted to hydrogen peroxide (H_2_O_2_) and the hydroxyl radical (OH•), which further stimulates oxidative stress and causes damage.

NADPH oxidases are the primary source of ROS. They oxidize NADPH to NADP^+^ and deliver electrons to O_2_, thus generating superoxide or H_2_O_2_. Nox/Duox family of NADPH oxidases has been reported to involve in ROS production during I/R injury by their facilitated activity (Wang et al., 2006; Simone et al., 2014). Nox2 has been a focus in I/R injury that occurs in stroke. Nox subunit-deficient mice and mice with apocynin (a Nox-2 inhibitor) pretreatment show remarkably decreased infarct volume and improved the clinical outcome of stroke (Chen et al., 2009; Jackman et al., 2009), suggesting that Nox-induced ROS plays a considerable role in I/R injury.

Besides immediately producing ROS, NADPH oxidases are also regulating ROS production by stimulating NO synthase system. NO, also known as endothelium-derived relaxing factor, is made from L-arginine by nitric oxide synthase (NOS) of three sources: Neuron NOS (nNOS), inducible NOS (iNOS), and endothelial NOS (eNOS). The role of NO is variable: It generally works as an anti-oxidant agent, but its interaction with the superoxide anion can lead to the formation of the peroxynitrite (ONOO^–^) (Marla et al., 1997). ROS created by NADPH oxidases can oxidize tetrahydrobiopterin (BH_4_), an essential cofactor that mediates eNOS activity. BH_4_ oxidation later induces the uncoupling of eNOS, resulting in decreased NO production and increased ONOO^–^ production from eNOS (Landmesser et al., 2003).

Mitochondria are the major site of oxidative stress generation, action, and injury. ROS can be generated from ETC. In ischemia, cellular stress can induce post-translational modifications of oxidative phosphorylation proteins in ETC, making them more sensitive to reoxygenation (Prabu et al., 2006). Disrupted ETC complexes can result in higher mitochondrial membrane potentials, positively associated with more ROS generation (Prabu et al., 2006). Enhanced oxidative stress can target mitochondria and further damage ETC, causing more ROS generation (Indo et al., 2007) subsequently. ROS from exogenous origins and mitochondrial ROS generation can lead to mitochondria DNA damage (Indo et al., 2007). In addition, too much oxidative stress can cellular damage or death (Figure 2).

#### Calcium Overload: Another Disturbance in Ischemia-Reperfusion Injury

In addition to oxidative stress caused by different sources, calcium overload, abnormally increased intracellular Ca^2+^ level is another major pathology that plays an important role in reperfusion injury. Anaerobic respiration in ischemia decreases intracellular pH; thus, the Na^+^/H^+^ exchanger (NHE) allows for the influx of Na^+^ to maintain the pH. NHE is generally inactivate during ischemia, but its activity can be increased during reperfusion, leading to a large Na^+^ influx (Allen and Xiao, 2003). A lower level of ATP in ischemia also weakens the activity of energy-dependent Na^+^ pump, resulting in a higher level of intracellular Na^+^. A study in 1987 suggested that the precedent sodium imbalance be a cause of calcium overload using an energy-repleted Na^+^ loading model (Grinwald and Brosnahan, 1987). Failing to return to normal Na^+^ balance upon oxygen restoration can promote the function of Na^+^/Ca^2+^ exchanger (NCX) that is sensitive to intracellular Na^+^ level, thus leading to higher Ca^2+^ influx. Calcium overload is also induced by elevated Ca^2+^ release and limited Ca^2+^ uptake from an internal source, including endoplasmic reticulum (ER) or Golgi apparatus (Chami et al., 2008). Promoted uptake of Ca^2+^ by mitochondria later occurs following cytosolic calcium overload (Brookes et al., 2004). Cytosolic and mitochondrial calcium overload can cause cellular damage in various ways, including disrupting mitochondrial function (Wang M. et al., 2015), promoting ROS production (Zhu et al., 2018), and inducing cell death (Boehning et al., 2004; Zhu et al., 2018) (Figure 2).

#### Mitochondria-Dependent Cell Death in I/R Injury

Cellular alternations, including increased oxidative stress and calcium overload, can lead to apoptosis with the involvement of mitochondria. This process is initiated by changes in mitochondrial membrane permeability controlled by mitochondrial permeability transition pore (mPTP). The activity of mPTP is likely to be mediated by mitochondrial matrix Ca^2+^ level, and the mitochondrial calcium overload resulting from cytosolic calcium overload can facilitate the opening of mPTP (Qian et al., 1999). ROS production during I/R injury, especially hydroxyl radicals and hydrogen peroxide, have also been found indispensable in mPTP opening (Assaly et al., 2012). Permeabilized membrane allows for the activation and insertion of pro-apoptotic Bcl-2 family members BAX and BAK into mitochondria membrane (Wei et al., 2000; Kirkland et al., 2002). This helps with transferring mitochondrial proteins including cytochrome c from mitochondria to the cytosol, followed by the interaction between cytochrome c and two cofactors, apoptotic protease activating factor 1 (APAF-1) and pro-caspase-9, to form the apoptosome, which eventually activates caspase–9-caspase-3 signaling cell death pathway with proteolytic events and DNA fragmentation (Broughton et al., 2009). This pathway is referred to as caspase-dependent apoptotic pathway.

Another cell death pathway, caspase-independent apoptosis, can be activated when cellular energy is running out (Daugas et al., 2000). Poly (ADP-ribose) polymerase-1 (PARP-1) is a nuclear enzyme that locates in the upstream of the pathway (Yu et al., 2002). ROS-induced DNA damage can trigger PARP-1 overactivation, in which NAD+ is used, thus depleting energy storage. Yu et al. (2002) also found that PARP-1 activation can lead to the release of its downstream target apoptosis-inducing factor (AIF, a mitochondrial flavoprotein) from mitochondrial intermembrane to nucleus, causing chromatin condensation and large-scale DNA fragmentation. Studies have indicated that AIF does not have a direct DNA fragmentation effect (Susin et al., 1999; Wang et al., 2002). Thus, it probably needs a downstream effector during this process. Studies have suggested that endonuclease G might interact with AIF and cause DNA fragmentation (Wang et al., 2002; Lee et al., 2005), though their interaction is still unclear. PARP-1-induced cell death is a unique cell death pathway. It generally exhibits characteristics of apoptosis, and it is also considered necrotic by some researchers since classic apoptosis is energy-dependent (Ha and Snyder, 1999).

### The Brain Is Susceptible to I/R Injury

I/R injury can occur in many organs and tissues, including the brain, heart, skeletal muscles, and kidney. Some common features are shared by I/R injury in these areas, including the elevated production of ROS, calcium overload, inflammation, and the opening of mPTP. Yet, organ-specific characteristics can affect the severity of I/R injury in different organs. Brain, the organ where irreversible damage occurs within 20 min after ischemia and a narrow time window (generally 3–4.5 h) can be given for reperfusion therapy, is considered very susceptible to I/R injury (Ordy et al., 1993). ROS in the brain is mostly generated from mitochondria rather than other enzymatic ROS sources as a metabolically active area. The brain accounts for more than 20% of total body oxygen consumption but with a relatively low antioxidative agent level compared with other organs, making it vulnerable to oxidative stress (Markesbery and Lovell, 2007; Damle et al., 2009; Kalogeris et al., 2012).

Moreover, accumulated labile iron in the brain can react with H_2_O_2_ to produce highly reactive •OH. This reaction stimulates the oxidation and peroxidation of massively accumulated polyunsaturated fatty acid in the brain, causing even more oxidative stress (Ferretti et al., 2008). Because of the brain’s susceptibility to I/R injury, finding targets to prevent reperfusion injury to the brain is significant in treating stroke.

### Extending the Therapeutic Time Window in Ischemic Stroke: Delayed Recanalization

Successful recanalization of the occluded vessel as early as possible has been widely accepted as the vital principle of AIS treatment. Unfortunately, for many years, most AIS patients were prevented from receiving effective recanalization therapy because of a narrow therapeutic window. In recent years, a series of clinical trials have indicated that delayed recanalization may still have benefits in ischemic brains during an expanded therapeutic window, up to more than 24 h, several days, and even more than 1 month after symptom onset [Reviewed by Kang et al. (2020)].

Clinically, advances in imaging techniques have allowed better characterization of brain tissue and vessel status in AIS. Markers of brain ischemia are instructed by perfusion-weighted imaging/diffusion-weighted imaging (PWI/DWI) mismatch and DWI/fluid-attenuated inversion recovery (DWI/FLAIR) mismatch on magnetic resonance imaging (MRI). MRI scanning with PWI or computed tomography (CT) perfusion (CTP) scanning shows different hypoperfusion levels. Given these developments, together with advances in intravascular interventional devices, expanding the recanalization time window in certain patients is possible.

Increasing randomized studies have demonstrated that delayed recanalization has beneficial effects on 90-day outcomes. Two high-quality, randomized controlled clinical trials (DAWN and DEFUSE 3) of endovascular mechanical thrombectomy reported that selective delayed recanalization based on imaging mismatch improved patients’ 90-day outcomes, even when performed at 16–24 h after symptom onset (Ragoschke-Schumm and Walter, 2018).

In summary, despite the risk of I/R injury, which might increase with the delayed time point for recanalization, delayed recanalization is still beneficial for a certain subtype of patients.

## Mitophagy in I/R Injury

### Mitophagy Is Activated Upon Ischemic Stroke

Enhanced mitochondrial fragmentation and fission were observed during both the ischemic phase and I/R injury. During OGD in rat cardiomyocyte cell line (H9C2 cells), massive mitochondria fragmentation was observed (Kim H. et al., 2011). Reoxygenation of mice cardiomyocyte cell line (HL1 cells) and neonatal primary cardiomyocytes show mitochondria fission (Ong et al., 2010; Disatnik et al., 2013). *In vivo* experiments using mice, 24-h left anterior descending permanent ligation model show consistent results (Kim H. et al., 2011). The brain tissue has a very similar situation as cardiomyocytes. Following OGD/reoxygenation, enhanced mitochondria fragmentation was observed in mice N2a neuroblastoma and primary rat neurons (Sanderson et al., 2015; Tang et al., 2016), accompanied by Opa1 processing and cytochrome C release. *In vivo* experiments in CA1 hippocampal neurons of rats have consistent results (Kumar et al., 2016).

**FIGURE 3:**
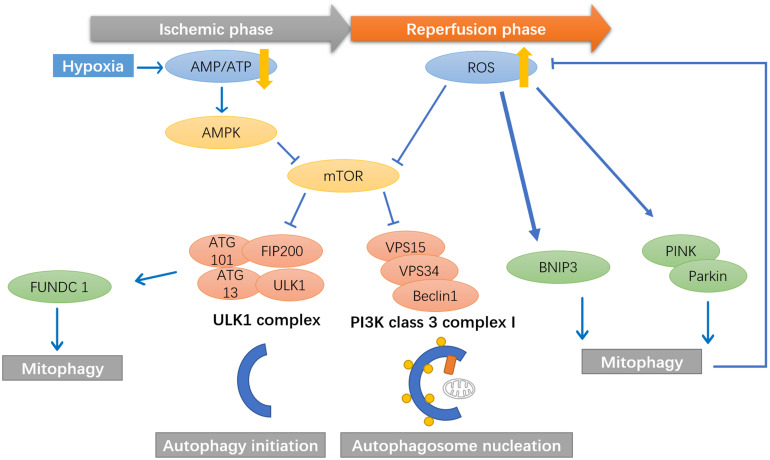
Mitophagy pathways are activated during ischemic/reperfusion through multiple signals. During the ischemic phase, ATP production rapidly decrease, which activates AMPK pathways to initiate autophagy. AMPK activates the phosphorylation of ULK1, which will then activate the class III PI3K complex (beclin 1, VPS34, and VPS15) that initiate the nucleation of phagophore. ULK1 has also been found to activate the FUNDC1 receptor, which may collaterally activate mitophagy. During the reperfusion phase, the mTOR pathways is inhibited by ROS signals, thereby promote the initiation and nucleation of the autophagosome. ROS has been found to activate BNIP3 and PINK1/Parkin mediated mitophagy in cerebral ischemia and reperfusion.

Later, researchers found that mitophagy pathways are activated during ischemic/reperfusion through multiple signals. During the ischemic phase, ATP production rapidly decreases, which activates AMPK pathways to initiate autophagy via direct activation of the ULK complex through phosphorylating of Ser 317 and Ser 777, or indirect activation of ULK through inhibiting the activity of mTOR, as mTOR suppresses Ulk1 activation by phosphorylating Ulk1 Ser 757 and disrupting the interaction between Ulk1 and AMPK (Kim J. et al., 2011; Zaha and Young, 2012). The activated ULK1 complex will then activate the class III PI3K complex (beclin 1, VPS34, and VPS15) that initiates the nucleation of phagophore (Kim J. et al., 2011). ULK1 has also been found to activate the FUNDC1 receptor, which may collaterally activate mitophagy (Kundu et al., 2008) (Figure 3).

During the reperfusion phase, the mechanistic target of rapamycin (mTOR) pathways is inhibited by ROS signals, thereby promoting the autophagosome’s initiation and nucleation (Alexander et al., 2010). ROS has been found to activate mitophagy via BNIP3, while high levels of BNIP3 can induce apoptosis (Scherz-Shouval et al., 2007). PINK1/Parkin-mediated mitophagy is also activated in brain damage induced by cerebral ischemia and reperfusion. Lan et al. (2018) found significant increases in PINK1 accumulation in the outer membrane of mitochondria. They increased Parkin/p62 mitochondrial translocation after reperfusion, along with upregulation of autophagy markers LC3B, Beclin1, LAMP-1, with a peak at 24 h (Figure 3).

### Mitophagy Exerts Its Protective Role Mainly During the Reperfusion Phase

Various studies have already demonstrated the protective role of enhanced mitophagy in attenuating brain injury after tMCAO in rats (Li et al., 2014; Di et al., 2015). However, whether mitophagy exerts its protective role during the ischemic phase of the reperfusion phase is unclear. Ischemia and reperfusion have different pathophysiology related to mitochondria. Thus, a series of studies have focused on distinguishing the ischemic and reperfusion phase. Intriguingly, mitophagy was identified to have a particular role during the reperfusion phase but may not the ischemic phase.

Inhibition of Drp1 was neuroprotective in response to OGD *in vitro* and transient focal ischemia *in vivo* (Grohm et al., 2012) and cardioprotective in cultured HL-1 cardiomyocytes subjected to OGD and reoxygenation (Dong et al., 2016). However, cardioprotection was only seen when inhibition of Drp1 was initiated as a pretreatment. When inhibiting Drp1 during reoxygenation, cell death was paradoxically exacerbated, indicating mitophagy is essential in protecting cells from reperfusion injury. In another study, researchers found that mitochondrial DNA level (which can be used as a marker for mitochondrial mass) does not decrease but significantly increase during the first few hours in the ischemic phase of stroke (Yin et al., 2008), which could indicate that mitochondrial biogenesis, but not mitophagy, is part of the post-stroke repair mechanisms during the ischemic phase. Kumar et al. (2016) also identified distinct phases of mitochondria during OGD and reoxygenation. During OGD occurs first round of fission, reoxygenation initially induces fusion but followed by massive fragmentation. These results have identified the distinct nature of the ischemic phase and reperfusion phase, in which mitophagy seems to have a unique protective role only in the reperfusion phase. However, given these results, which monitor mitophagy accurately to different phases, a fuller picture of mitophagy dynamic in the ischemic/reperfusion process is emerging, but still with some obscurity. Thus, a more comprehensive and accurate evaluation of different time points is required to fully explain the role of mitophagy in both ischemic and reperfusion injuries.

### The Interplay Between Mitophagy and IR Injury

#### Mitochondria-Dependent Cell Death in I/R Injury

During both the ischemic phase and IR injury, mitochondrial-dependent cell apoptosis is one of the critical events defining cell fate. The BCL-2 family proteins, which is a significant regulator of outer mitochondrial membrane permeability and play an essential role in the intrinsic apoptotic pathway (Chao and Korsmeyer, 1998), provide the mitochondria for the function of “sensing” apoptotic stress. The BCL-2 family has been classified into two groups: anti-apoptotic proteins (including Bcl-2, Bcl-xL, and Bcl-w) and pro-apoptotic proteins (including Bax, Bak, etc.) (Oltvai et al., 1993). Under stress conditions such as ischemic insults and IR injury, the anti-apoptotic protein Bcl-2 is phosphorylated and releases Beclin1 to activate autophagy and mitophagy. Bcl-2 prevents the release of pro-apoptotic proteins by maintaining the integrity of the mitochondrial membrane. This process inhibits cell apoptosis (Praharaj et al., 2019). However, pro-apoptotic BCL-3 subgroup proteins are also found to be upregulated after ischemic stroke, which induces the release of cytochrome c from mitochondria intermembrane into the cytosol. Cytochrome c interacts with the protein cofactor Apaf-1 and procaspase-9 form apoptosome (Gibson and Davids, 2015). Thus, ischemia and IR injury may elicit complex apoptotic effects involving mitochondria. The activities of anti- and pro-apoptotic proteins are correlated with the ROS concentration.

#### Mitophagy and Ca^2+^ Overload

Calcium overload is an essential pathology in I/R injury as it promotes the opening of mPTP, which activates apoptotic factors such as the Bcl-2 family and leads to cell apoptosis eventually. Recent evidence suggests a link between mitophagy and Ca^2+^ regulation. Many mitophagic proteins are also involved in mitochondrial calcium regulation. For instance, in PINK1/Parkin mediated mitophagy, many Ca^2+^ sensing proteins are ubiquitinated, which disturb the function as a Ca^2+^ buffering system, such as MFNs (de Brito and Scorrano, 2008; Gegg et al., 2010; Filadi et al., 2015), DRP1 (Cereghetti et al., 2008; Wang et al., 2011), VDAC1 (Gincel et al., 2001; Geisler et al., 2010) and BCL2 (Rong et al., 2008; Chen et al., 2010). Moreover, PINK1 phosphorylates mitochondrial rho GTPase 1 (RHOT1), which is an essential regulator of the Ca^2+^ sensitive characteristics in ER phospholipid exchange (Kornmann et al., 2011) and mitochondrial dynamics (Saotome et al., 2008). Parkin mediates the ubiquitination and degradation of MICU-1, which also affects the stability of MICU2 (Matteucci et al., 2018). Additionally, depending on the calcium level in the ER, Ca^2+^ can be either pro- or anti-autophagic (Cárdenas et al., 2010).

#### Mitophagy and Oxidative Stress

Oxidative stress is one of the most critical pathological brain damage processes in acute ischemic stroke (Chamorro et al., 2016). ROS and reactive nitrogen species (RNS) are crucial mediators in cerebral ischemia-reperfusion injury. ROS/RNS at a low level is beneficial for facilitating adaptation to stress as a redox signaling, whereas ROS/RNS at a high level is deleterious (Scialò et al., 2016). ROS and RNS have mainly been generated from the electron transport chain (ETC) complexes I and complex III in mitochondria, and excessive ROS/RNS primarily induces mitochondria injury. Under normal condition, less than 10% of oxygen receive electrons to generate superoxide in ETC. However, upon IR injury, the ischemic brains produce a large amount of NO and superoxide anion (O_2_^–^) simultaneously. The rapid reaction of NO and O_2_^–^ leads to the formation of ONOO^–^, a representative/critical cytotoxic factor for both oxidative and nitrosative stress (Marla et al., 1997). ONOO^–^ is a highly active cytotoxic molecule to aggravate neuronal damage, disrupt the blood–brain-barrier (BBB) integrity, and mediate hemorrhagic transformation via triggering numerous cellular signaling cascades, which represents a vital pathogenic mechanism in ischemic stroke (Marla et al., 1997). Mitophagy has regulatory effects on oxidative stress since damaged mitochondria will lead to the release of more ROS. ROS can directly induce mitophagy (Wang et al., 2012), which has been shown beneficial in various disease conditions as mitophagy attenuates oxidative stress. However, multiple studies have demonstrated that ONOO^–^ induces excessive mitophagy activation by tyrosine nitration of Drp1 and mediates mitochondrial recruitment of Drp1, thus aggravating cerebral I/R injury (Feng et al., 2017). This raises the question of whether mitophagy is beneficial or detrimental in response to I/R injury and will be discussed later. Indeed, ONOO^–^-mediated mitophagy is suspected to be excessive and could be a crucial therapeutic target for IR injury.

#### Mitophagy and Inflammation

Anti-inflammation is another function of mitophagy. Upon mitochondria dysfunction, leaked mitochondrial ROS can activate inflammasomes, contributing to inflammation (Murakami et al., 2012). Mitophagy has shown a positive effect in kidney disease, ischemic stroke, and I/R injury. For instance, upon encountering I/R injury, kidney tubules rich in mitochondria will enhance autophagy (including mitophagy) to protect cells from ROS-triggered inflammatory response (Kimura et al., 2011; Zhou et al., 2011). In ischemic stroke, overexpression of the ATF4 gene can enhance mitophagy and inhibit NLRP3 inflammasome-mediated inflammatory response (He et al., 2019). Inducing Parkin-dependent mitophagy can improve inflammatory response and prevent cell death in myocardial I/R injury (Yao et al., 2019).

### Therapeutic Potential of Mitophagy Regulation in I/R Injury

Recent years boosts the research of mitophagy in cerebral ischemia and reperfusion, most of them demonstrating the protective role of mitophagy. However, excessive mitophagy, which is probably induced by certain oxidative stress conditions, has also been shown to be deleterious in cerebral ischemia and reperfusion processes. In pre-clinical studies, several therapeutic agents have been proposed to ameliorate cerebral I/R injury, either through enhancing or inhibiting certain types of mitophagy. These mitophagy-related interventions could be proposed as adjunctive approaches for ischemic stroke management.

Studies investigating the role of mitophagy in I/R injury after 2018 are summarized as follows and in Table 1. Earlier studies have been reviewed by Anzell et al. (2018) and Guan et al. (2018). The role of autophagy and potential strategies in ischemic stroke is also comprehensively summarized in a recent review (Ajoolabady et al., 2021).

**TABLE 1:** Summary of Part 3.4 Therapeutic potential of mitophagy in I/R injury.

Therapy	Mitophagy	Other pathways involved	Reference
Electroacupuncture	Activate PINK/Parkin-mediated mitophagy	PI3K/Akt signaling pathway	Wang et al., 2019; Mao et al., 2020
ATF4 overexpression	Activate PINK/Parkin-mediated mitophagy	Inhibit NLRP3 inflammasome-mediated inflammatory response	He et al., 2019
Curcumin	Activate	/	Wang and Xu, 2020
GeB	Activate PINK/Parkin-mediated mitophagy	/	Wu et al., 2020
GeX1 and McX	Activate PINK/Parkin-mediated mitophagy	/	Xiang et al., 2020
ANNAO	Activate	/	Zhang et al., 2020a
A sphingosine kinase 2-mimicking TAT-peptide	Activating BNIP3-mediated mitophagy	/	Chen et al., 2020
Enhance retrograde transport of mitochondria	Assisted activation	/	Zheng et al., 2019a,b
Naringin	Inhibit	Inhibit ONOO^–^ mediated mitophagy	Feng et al., 2018
Rehmapicroside	Inhibit	Inhibit ONOO^–^ mediated mitophagy	Zhang et al., 2020b

#### Protective Role of Enhanced Mitophagy in IR Injury

Zhang and Yu (2018) found that after reperfusion, NR4A1 was significantly elevated in the brain tissue, inhibiting the activation of protective mitophagy through the MAPK–ERK–CREB signaling pathway. Genetic ablation of NR4A1 reduced the cerebral infarction area and neuronal apoptosis. As demonstrated by functional study, NR4A1 modulated cerebral IR injury by inducing mitochondrial damage. Higher NR4A1 promoted mitochondrial potential reduction, aggravated cellular oxidative stress, and initiated caspase-9-dependent apoptosis. Mechanistically, NR4A1 induced mitochondrial damage by disrupting Mfn2-related mitophagy. Knockdown of NR4A1 reversed mitophagic activity, sending a prosurvival signal for mitochondria in the setting of cerebral IR injury (Zhang and Yu, 2018).

Electroacupuncture (EA) has been shown effective in treating ischemic stroke. Recently, Wang et al. (2019) demonstrated that EA ameliorates nitro/oxidative stress-induced mitochondrial functional damage and reduces the accumulation of damaged mitochondria via Pink1/Parkin-mediated mitophagy clearance to protect cells against neuronal injury in cerebral I/R. Later, Mao et al. (2020) also found EA pretreatment has a protective effect on cerebral I/R injury through promoting mitophagy, based on the results that the number of autophagosomes, FUNDC1, p62, and the ratio of LC3-II/I were significantly increased. Still, mitochondrial membrane potential and autophagy-related protein p-mTORC1 significantly decreased in the I/R group (Mao et al., 2020).

He et al. (2019) showed that ATF4 overexpression induced by AAV was protective against cerebral I/R injury by upregulating Parkin expression, enhance mitophagy activity and inhibit NLRP3 inflammasome-mediated inflammatory response.

A series of natural compounds have been found to possess the ability to promote mitophagy and attenuate I/R injuries. Curcumin is a complex extracted from the traditional edible herb which can protect neurons in rats after brain I/R injury (Wang and Xu, 2020). Curcumin reduced the levels of ROS while increased state 3 respiration to prevent the impairment of mitochondrial function from cerebral I/R. Furthermore, curcumin enhanced the co-localization of LC3B and mitochondrial marker VDAC1, the ratio of LC3-II to LC3-I, suggesting the protective role of curcumin exerts through enhancing mitophagy. Wu et al. (2020) found that garciesculenxanthone B (GeB), a new xanthone compound from *Garcinia esculenta*, can promote the PINK1-Parkin-mediated mitophagy pathway and protects the brain from I/R injury. Treatment with GeB dose-dependently promoted the degradation of mitochondrial proteins Tom20, Tim23, and MFN1 in YFP-Parkin HeLa cells and SH-SY5Y cells. GeB stabilized PINK1 and triggered Parkin translocation to the impaired mitochondria to induce mitophagy, and these effects were abolished by knockdown of PINK1. *In vivo* experiments demonstrated that GeB partially rescued ischemia-reperfusion-induced brain injury in mice. Another two natural compounds named Gerontoxanthone I (GeX1) and Macluraxanthone (McX), were also screened out to possess the ability to enhance mitophagy (Xiang et al., 2020). GeX1 and McX directly stabilized PINK1 on the outer membrane of the mitochondria and then recruited Parkin to mitochondria, suggesting that GeX1 and McX mediate mitophagy through the PINK1-Parkin pathway. GeX1 and McX treatment decreased cell apoptosis and the ROS level in an IR injury model in H9c2 cells. Additionally, a tablet derives from Chinese classical prescriptions of Angong Niuhuang Pills with modified compositions has also been shown to attenuate cerebral I/R injury by improving mitophagy and mitochondrial quality control (Zhang et al., 2020a). The tablets elevated the ratio of Bcl-2/Bax, inhibited apoptosis, decreased the infarction volume, and improved the MCAO rats’ behavioral performance.

Although the precise molecular regulatory network of these natural compounds has not been fully addressed, considering the advantages of natural compounds such as low toxicity and safe pharmacokinetic profiles including high utility rate and effective elimination, drugs derived from natural compounds are of high translational value.

Chen et al. (2020) demonstrated that a sphingosine kinase 2-mimicking TAT-peptide protects neurons against ischemia-reperfusion injury by activating BNIP3-mediated mitophagy. sphingosine kinase 2 (SPK2) interacts with Bcl-2 via its BH3 domain, activating autophagy or mitophagy by inducing the dissociation of Beclin-1/Bcl-2 or Bcl-2/BNIP3 complexes and protects neurons against ischemic injury.

Different from cardiac muscle, the long axon is a highly distinct morphology of neurons, where located more than half of total mitochondria content in neurons (Nafstad and Blackstad, 1966). However, under stress conditions, autophagy and mitophagy events are concentrated in the soma (Maday and Holzbaur, 2014), but not in exon, evidenced by concentrated autophagosomes and lysosomes in the soma (Farías et al., 2017). Thus, it is unclear how mitochondria in distal axons are cleared in ischemic neurons (Ashrafi et al., 2014). Zheng et al. (2019a,b) have identified unique mitochondria movement in neurons, from axon to soma for degradation. Upon oxygen and glucose deprivation-reperfusion, axonal mitochondria showed loss of anterograde motility but increased retrograde motion upon reperfusion, meaning axonal mitochondria are transported to the neuronal soma for degradation. Anchoring of axonal mitochondria by syntaphilin blocked neuronal mitophagy and aggravated injury. Conversely, induced binding of mitochondria to dynein reinforced retrograde transport and enhanced mitophagy prevent mitochondrial dysfunction and attenuate neuronal damage. Therefore, regulating mitochondria motility in neurons would be another direction for enhancing mitophagy and attenuating I/R neuronal injury.

#### ONOO^–^ Induces Deleterious Mitophagy

Feng et al. (2018) found that naringin, a natural antioxidant, could inhibit ONOO^–^ mediated mitophagy activation and attenuate cerebral I/R injury. Naringin possessed strong ONOO^–^ scavenging capability and inhibited the production of superoxide and nitric oxide in IR injury conditions. Naringin inhibited the expression of NADPH oxidase subunits and iNOS in rat brains subjected to 2 h ischemia plus 22 h reperfusion. Naringin can cross the blood-brain barrier, decreases neurological deficit score, reduces infarct size, and attenuates apoptosis in the ischemia-reperfused rat brains. Furthermore, naringin decreased the ratio of LC3-II to LC3-I in mitochondria. It inhibited the translocation of Parkin to the mitochondria, suggesting naringin prevents the brain from I/R injury via attenuating ONOO^–^-mediated excessive mitophagy.

Rehmapicroside, a natural compound from a medicinal plant, can inhibit ONOO^–^-mediated mitophagy activation (Zhang et al., 2020b). *In vitro*, rehmapicroside reacted with ONOO^–^ directly to scavenge ONOO^–^, decreased O2^–^ and ONOO^–^, up-regulated Bcl-2 but down-regulated Bax, Caspase-3 and cleaved Caspase-3, and down-regulated PINK1, Parkin, p62 and the ratio of LC3-II to LC3-I in the OGD/RO-treated PC12 cells. *In vivo*, rehmapicroside suppressed 3-nitrotyrosine formation, Drp1 nitration as well as NADPH oxidases and iNOS expression in the ischemia-reperfused rat brains; it also prevented the translocations of PINK1, Parkin, and Drp1 into the mitochondria for mitophagy activation; finally, rehmapicroside ameliorated infarct sizes and improved neurological deficit scores in the rats with transient MCAO cerebral ischemia.

Deng et al. (2020) demonstrate that lncRNA SNHG14 promotes OGD/R-induced neuron injury by inducing excessive mitophagy via miR-182-5p/BINP3 axis in HT22 mouse hippocampal neuronal cells. SNHG14 and BNIP3 were highly expressed, and miR-182-5p was down-regulated in the OGD/R-induced HT22 cells. OGD/R-induced HT22 cells exhibited an increase in apoptosis. SNHG14 overexpression promoted apoptosis and the expression of cleaved-caspase-3 and cleaved-caspase-9 in the OGD/R-induced HT22 cells. Moreover, SNHG14 up-regulation enhanced the expression of BNIP3, Beclin-1, and LC3II/LC3I in the OGD/R-induced HT22 cells. Furthermore, SNHG14 regulated BNIP3 expression by sponging miR-182-5p. MiR-182-5p overexpression or BNIP3 knockdown repressed apoptosis in OGD/R-induced HT22 cells, which was abolished by SNHG14 up-regulation.

Taken together, inhibiting the ONOO^–^-mediated excessive mitophagy activation exerts neuroprotective effects, with several potential drug candidates being discovered to attenuate cerebral IR injury. Although most studies have demonstrated the possible protective effects of mitophagy upon IR injury, mitophagy is a double-edged sword and requires more studies to test its clinical potential.

## Discussion and Future Perspectives

In the treatment of ischemic stroke, reperfusion with thrombolysis and thrombectomy are key to restoring blood flow and improving patient outcomes. However, restoration of blood flow in patients with AIS may result in secondary reperfusion injury. Resupply of oxygen can cause the overactivation of enzymes and pumps that are previously inhibited by ischemia-induced ATP deficiency, thus resulting in the boost of reactive oxygen species (ROS) production and altering calcium homeostasis in both cytoplasm and mitochondria. Such alternations can induce mitochondrial DNA damage and promote the opening of mPTP, which triggers apoptosis-related factors and result in cell death.

Mitophagy is an essential cellular process that maintains mitochondrial quality. While IR injury primarily induces mitochondrial dysfunction and leads to dysregulation of oxidative stress, calcium homeostasis, and cell apoptosis, regulation of mitochondria dynamics (fission and fusion) and activation of a moderate level mitophagy can contribute to the adjustment of cellular mitochondria quality. Identifying and targeting mitophagy-related pathways molecules may benefit certain subsets of patients with ischemic stroke. And some mitophagy regulators discussed above have already shown great potential in clinical application. For example, for acute ischemic stroke patients, taking some herbal agents prior to reaching the hospital, and more importantly, during the long recovery stage, may provide vital neuroprotection effects and lead to a better prognosis. To address the clinical potential of mitophagy, further elucidation of mitophagy and its crosstalk mechanism under stroke conditions is required; further discovery of therapeutic targets and drug development for manipulating the mitophagy pathways are needed.

## Author Contributions

CS and TZ: conceptualization and supervision. LS, QG, YY, and CS: literature search and writing. LS, QG, and YY: figure sketching. CR, SX, ZZ, and TZ: editing and formatting. TZ: funding acquisition. All authors have read and agreed to the published version of the manuscript.

## Conflict of Interest

The authors declare that this study received funding from Ningbo Guangyuan Zhi Xin Biotechnology Co., Ltd. The funder was not involved in the study design, collection, analysis, interpretation of data, the writing of this article or the decision to submit it for publication.
